# Optimizing the Measurement of Colostrum Antibody Concentrations for Identifying BVDV Persistently Infected Calves

**DOI:** 10.3390/vetsci2010026

**Published:** 2015-03-09

**Authors:** Caitlin J. Jenvey, Michael P. Reichel, Sasha R. Lanyon, Peter D. Cockcroft

**Affiliations:** School of Animal and Veterinary Sciences, The University of Adelaide, Roseworthy Campus, Roseworthy, SA 5371, Australia; E-Mails: michael.reichel@adeliade.edu.au (M.P.R.); sasha.lanyon@adelaide.edu.au (S.R.L.); peter.cockcroft@adelaide.edu.au (P.D.C.)

**Keywords:** BVDV, colostrum, ELISA, PI calves

## Abstract

Colostrum contains substantially higher concentrations of immunoglobulins compared to serum, which may help to improve the utility of diagnostic tests. The aim of this study was to determine the diagnostic value of colostrum antibody concentrations in identifying Bovine Viral Diarrhoea Virus (BVDV) PI (persistently infected) calf carrying beef heifers following an experimental infection. Colostrum was collected within 12 hours of parturition and tested in undiluted, 1:5, 1:10, 1:100, 1:200, and 1:500 dilutions using an enzyme-linked immunosorbent assay (ELISA) for BVDV antibody. Cows were determined to be carrying a PI calf based on positive quantitative Real Time-Polymerase Chain Reaction and antigen ELISA result on pre-colostral serum and ear notch samples collected from their calf. The median ELISA sample-to-positive (S/P) ratio for colostrum collected from heifers that carried a PI calf were significantly higher than the median ELISA S/P ratio for colostrum collected from heifers that did not carry a PI calf at dilutions of 1:100, 1:200, and 1:500. This study provides further evidence for increased antigenic stimulation *in utero* by the BVDV viraemic PI calf, which can also be identified with 100% diagnostic sensitivity when using 1:500 dilution colostrum.

## 1. Introduction

Colostrum contains high concentrations of maternal antibodies that are selectively transferred into the mammary gland from the serum of the dam prior to calving. Concentrations of antibodies in bovine colostrum can be up to five [1] to 10 times [2] greater when compared to serum. Due to these high antibody concentrations, colostrum could improve the utility of diagnostic tests by increasing the magnitude of detectable differences between cohorts of individual animals, including, but not limited to, cows infected with Bovine Viral Diarrhoea Virus (BVDV). Dams that are infected with BVDV between 40 and 120 days of gestation may produce persistently infected (PI) offspring that test negative for BVDV-specific antibodies [3]. These PI animals are important reservoirs of infection due to their high persistent levels of viral excretion and infectivity for susceptible cattle [4]. There is evidence to suggest that the BVDV antibody concentration of dams carrying PI calves are elevated during late gestation when compared to previously infected dams carrying non-PI calves [5,6,7], but this difference has not been convincing enough to be utilised in practice (early detection of PI calves). These studies found that, on average, the antibody levels of cows carrying PI calves and cows not carrying PI calves tended to overlap, and although samples should be collected in the last few months of gestation, there is larger variation in antibody levels between these two groups at parturition compared with early pregnancy [5,6,7]. There is little research into whether this increase in BVD specific antibody concentrations may also be found in colostrum collected from dams carrying PI calves. It is possible that concentrations found in colostrum may be quantitatively greater compared to serum, thus also allowing identification of the PI-carrying dam using colostrum. The aim of this study was to determine the diagnostic value of colostrum BVDV antibody concentrations in identifying PI calves following an experimental infection of a group of beef heifers.

## 2. Methods

### 2.1. Heifers

Twelve beef heifers were experimentally infected with BVDV-type 1c by co-mingling with a PI dairy cow from day 90, post artificial insemination [8]. All cows seroconverted within 28 days of exposure (antibody enzyme-linked immunosorbent assay), at which time the PI cow was removed from further direct or indirect contact with the heifers. Heifers were determined to have been carrying a PI calf based on positive quantitative real-time polymerase chain reaction and antigen ELISA result on serum and ear notch samples collected from their calf prior to colostrum ingestion.

### 2.2. Antibody ELISA

Colostrum was collected within 12 hours of calving and stored at −80 °C until testing could be performed. The colostrum samples were tested as undiluted samples, as well as at dilutions of 1:5, 1:10, 1:100, 1:200, and 1:500. Samples were diluted using the diluent supplied with the ELISA kit and tested in triplicate using the IDEXX BVDV Total Ab Test. Results were expressed as sample-to-positive (S/P) ratios.

### 2.3. Statistical Analysis

A Mann-Whitney U test was performed to identify significant differences between colostrum from heifers carrying PI calves and heifers that did not carry PI calves at each dilution, with a *p*-value < 0.01 considered significant. A two-graph receiver operating characteristic analysis (TG-ROC) was performed to determine DSe, DSp, and 95% confidence interval (CI) of the antibody ELISA for the detection of a PI calf for each dilution, or no dilution. The diagnostic sensitivity (DSe) and diagnostic specificity (DSp) were calculated using the following equations:

(1) $$\text{DSe}=\frac{\text{Number of heifers carrying PI calves that tested ELISA positive}}{\text{Number of heifers carrying PI calves}}\;\times 100$$

(2) $$\text{DSp}=\;\frac{\text{Number of heifers carying non}-\text{PI calves that tested ELISA negative}}{\text{Number of heifers carrying non}-\text{PI calves}}\;\times 100$$

## 3. Results

Of the twelve beef heifers that were experimentally infected, the calves of three heifers tested virus positive by RT-PCR and antigen positive by ELISA in their serum prior to colostrum ingestion. The calves from the remaining eight heifers tested virus negative and antigen negative and one heifer aborted her fetus 253 days post-AI. The median ELISA S/P ratio for colostrum collected from heifers carrying a PI calf were significantly higher compared to colostrum collected from heifers that were not carrying a PI calf for dilutions of 1:100 (*p* < 0.05), 1:200 (*p* < 0.01), and 1:500 (*p* < 0.01) (Table 1). For undiluted and 1:5 diluted colostrum, the median ELISA S/P ratio for colostrum samples collected from heifers carrying and not carrying PI calves did not differ significantly (Table 1).

**Table 1:** Median (Range) optical density value for colostrum collected from cows carrying and not carrying PI calves for dilutions of 0, 1:5, 1:100, 1:200, and 1:500.

Dilution	Heifers carrying PI calf	Heifers not carrying PI calf
0	2.08 (1.56–2.13) ^a^	2.22 (1.63–2.52) ^a^
1:5	2.55 (2.58–2.51) ^b^	2.55 (2.38–2.66) ^b^
1:100	2.21 (2.31–2.15) ^c^	1.81 (1.14–2.19) ^d^
1:200	1.98 (2.07–1.89) ^e^	1.50 (0.85–1.85) ^f^
1:500	1.81 (1.83–1.67) ^g^	1.00 (0.37–1.60) ^h^

^a^ Different superscript letters in the same row are statistically different (Mann-Whitney U Test).

**Figure 1:**
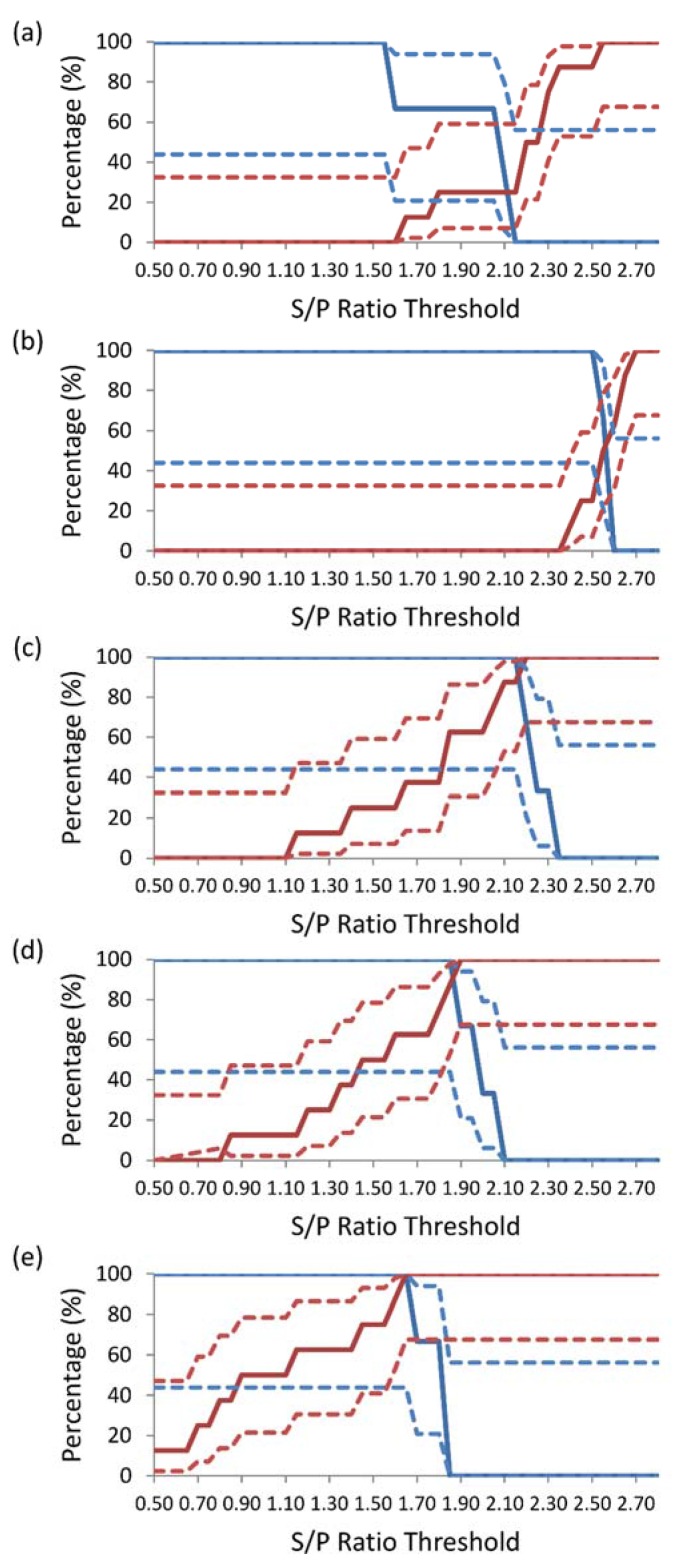
Two-graph receiver operating characteristic analysis of (**a**) undiluted colostrum, (**b**) 1:5 dilution colostrum, (**c**) 1:100 dilution colostrum, (**d**) 1:200 dilution colostrum, and (**e**) 1:500 dilution colostrum, using a commercial enzyme-linked immunosorbent assay for the detection of antibodies specific to BVDV. Diagnostic sensitivity (solid blue line) ± 95 percent confidence interval (dotted blue line) was calculated using sample-to-positive (S/P) ratio results for colostrum collected from heifers carrying PI calves (n = 3) and diagnostic specificity (red line) ± 95 per cent confidence interval (dotted red line) was calculated using S/P ratio results for colostrum collected from heifers carrying non-PI calves (n = 8).

The TG-ROC for DSe and DSp for each dilution are shown in Figure 1. The value of the DSe and DSp at the intercept increased with the dilution of the sample, reaching 100% at a colostrum dilution of 1:500. The DSe, DSp, and S/P threshold at the intercept for each dilution were: 33% at an S/P ratio threshold of 2.10 for undiluted colostrum (Figure 1a), 56% at a 1:5 dilution colostrum at an S/P ratio threshold of 2.60 (Figure 1b), 93.9% at a 1:100 dilution colostrum at an S/P ratio threshold of 2.20 (Figure 1c), 93.9% at a 1:200 dilution colostrum at an S/P ratio threshold of 1.90 (Figure 1d), and 100% at a 1:500 dilution colostrum at an S/P ratio threshold of 1.65 (Figure 1e).

## 4. Discussion

The purpose of this study was to determine the diagnostic value of colostrum BVDV antibody concentrations in identifying PI calves following an experimental infection of beef heifers. Previous studies [5,6,7] have demonstrated higher BVDV antibody concentrations in serum collected from seropositive cows carrying PI calves when compared to cows that do not carry a PI calf. However, there is little research into whether this same claim can be made with regards to colostrum samples. These previous studies also demonstrated some overlapping of antibody levels from cows that carried a PI calf and cows that did not carry a PI calf [5,6,7]. This result was also demonstrated in this study as no significant differences were observed in the median ELISA S/P ratios for heifers that carried a PI calf and heifers that did not carry a PI calf when colostrum was tested undiluted or diluted 1:5. However, the median ELISA S/P ratio for colostrum collected from heifers that carried a PI calf were significantly higher compared to the median ELISA S/P ratio for colostrum collected from heifers that did not carry a PI calf for dilutions of 1:100, 1:200 and 1:500. In addition, DSe and DSp of the ELISA was 100% when using colostrum diluted at 1:500. The results of this study indicate that colostrum, when diluted at 1:500, could be used to identify PI calves immediately following calving. It is possible that further dilution of the serum collected in these previous studies (1:10 [6] and 1:100 [6,7]) may have further increased the differences in antibody levels observed between cows that carried a PI calf and cows that did not carry a PI calf, thus, reducing the overlapping of antibody levels observed between the two groups.

This study provides further evidence for increased antigenic stimulation *in utero* by the BVDV viraemic PI calf. This study does not make any recommendations regarding the replacement of other PI detection methods with colostrum antibody testing, but rather, this study highlights the fact that colostrum can be used for PI calf detection and may be used in conjunction with other methods of detection.

## 5. Conclusions

The improvement in DSe observed in this study when colostrum was diluted, highlights the ability to improve the diagnostic utility of a test when disease-specific antibody concentrations (*i.e.*, BVDV antibody concentrations) supersede the ability of the ELISA to measure beyond the maximal S/P threshold. Additionally, colostrum may also benefit tests that demonstrate poor test characteristics, such as the poor diagnostic sensitivity experienced when testing for Johne’s disease. This approach, while tested here in beef heifers, could most readily be applied in dairy cows, where colostrum is routinely and easily collected by the producer.

## References

[1] Beer A., Billingham R., Head J. (1974). The immunologic significance of the mammary gland. J. Investig. Dermatol..

[2] Baurucker C., Burkett A., Magliaro-Macrina A., Dechow C. (2010). Colostrogenesis: Mass transfer of immunoglobulin G1 into colostrum. J. Dairy Sci..

[3] Grooms D. (2004). Reproductive consequences of infection with bovine viral diarrhoea virus. Vet. Clin. North Am. Food Anim. Pract..

[4] Lindberg A., Alenius S. (1999). Principles for eradication of bovine viral diarrhoea virus (BVDV) infections in cattle populations. Vet. Microbiol..

[5] Brownlie J., Hooper L., Thompson I., Collins M. (1998). Maternal recognition of foeatl infection with bovine virus diarrhoea virus (BVDV)—The bovine pestivirus. Clin. Diagn. Virol..

[6] Lindberg A., Niskanen R., Gustafsson H., Bengtsson B., Baule C., Belak S., Alenius S. (2001). Prenatal diagnosis of persistent Bovine Viral Diarrhoea Virus (BVDV) infection by detection of viral RNA in fetal fluids. Vet. J..

[7] Stokstad M., Niskanen R., Lindberg A., Thoren P., Belak S., Alenius S., Loken T. (2003). Experimental infection of cows with Bovine Viral Diarrhoea Virus in early pregnancy—Findings in serum and foetal fluids. J. Vet. Med. Series B.

[8] Lanyon S., Sims S., Cockcroft P., Reichel M. (2014). Comparison of serum, ear notches, and nasal and salival swabs for Bovine Viral Diarrhoea Virus antigen detection in colostrum-fed persistently infected (PI) calves and non-PI calves. J. Vet. Diagn. Investig..

